# Uniform in Bandwidth Consistency of the *L*^1^-Modal Regression Estimator for High-Dimensional Data

**DOI:** 10.3390/e28050558

**Published:** 2026-05-15

**Authors:** Fatimah A. Almulhim, Mohammed B. Alamari, Ali Laksaci

**Affiliations:** 1 Department of Mathematical Sciences, College of Science, Princess Nourah bint Abdulrahman University, P.O. Box 84428, Riyadh 11671, Saudi Arabia; faalmulhim@pnu.edu.sa; 2 Department of Mathematics, College of Science, King Khalid University, Abha 62223, Saudi Arabia; alikfa@kku.edu.sa

**Keywords:** nonparametric regression, functional data, stochastic consistency, quantile regression, shannon’s entropy, unformity in bandwidth, bandwidth parameter

## Abstract

We propose a new nonparametric estimator of the conditional mode in a regression framework where the covariates are functional in nature. The estimator is constructed through a quantile regression approach, which provides a robust alternative to classical density-based procedures. It is well documented that employing the $L^{1}$-structure in quantile regression, the estimation procedure improves robustness properties, particularly resistance to outliers and heavy-tailed error distributions. This feature makes the $L^{1}$ estimation of the conditional mode more stable and reliable in complex and high-variability functional data settings. The main objective of this paper is to establish strong consistency, with explicit convergence rates, for the associated kernel estimators, uniformly over a range of bandwidth parameters. The latter is developed under general regularity conditions involving the concentration distribution of the functional regressor, smoothness assumptions on the structural components of the model, and entropy conditions ensuring adequate control of the functional class complexity. Uniformity in bandwidth is essential both from a theoretical and practical issues, as it guarantees stability of the estimator under data-driven smoothing parameter selection. Beyond its theoretical contribution, this paper has direct implications for applied statistics. Specifically, it provides mathematical support for the automatic bandwidth selection procedures in the high-dimensional data context. Furthermore, the main theoretical novelty is highlighted through simulation experiments and applications to real data.

## 1. Introduction

**Research Problem and Related Literature**. Modeling the dependence between a scalar outcome and a functional random element is a fundamental issue in nonparametric functional data analysis. Over the past decades, considerable attention has been devoted to this topic (see, for instance, [1], among others). Statistical issues arising from high-dimensional structure of functional data have become a central research theme, as reflected in several special issues devoted to this framework in leading statistical journals (e.g., [2,3,4]).

The present investigation contributes to the field of nonparametric high-dimensional data analysis (NHDDA). Since the publication of [5], nonparametric modeling of functional data has emerged as one of the most dynamic and rapidly evolving areas of statistical research. In this context, the robust estimation of regression functions has attracted increasing attention, as it provides a reliable and flexible alternative to classical methodologies that may be sensitive to atypical observations or model deviations. The introduction of robust procedures into nonparametric functional statistics dates back to [6], who established the almost complete convergence of robust regression estimators for functional covariates under the assumption of independent and identically distributed observations. Motivated by its strong predictive performance, significant advances have been achieved in the development of robust nonparametric methods for functional regression. Early influential work [7], while more recent developments and comprehensive treatments of the subject can be found in [8,9], and the references therein. In parallel the estimation of the conditional mode has attracted significant interest in the NHDDA setting. Particularly, the functional version of the Nadaraya–Watson (NW) estimator for the conditional mode was first introduced in [10] as foundational study, where almost complete consistency was established by characterizing the estimator as the maximizer of the conditional density. Ref. [11] has extended this result to dependent functional processes. The asymptotic distribution of the NW-based functional conditional mode estimator was later derived under the i.i.d. framework, and further generalized to strongly mixing functional time series under fractal-type conditions (see [12]). Additional theoretical results on functional mode estimation, such as the $L^{p}$-convergence of spatial NW-type estimators, are provided in Ref. [13]. Asymptotic results related to the robust estimation of the conditional mode function can be found in [14].

**Main Contributions of This Study**. The majority of previous studies have focused on kernel-based methods for fitting nonparametric modal regression. These procedures are well known for their adequate asymptotic properties, which critically depend on the choice of the smoothing parameter. In general, reducing the bandwidth increases the estimator’s sensitivity to data sparsity in the functional covariate space, leading to higher variance. In contrast, an excessively large bandwidth can increase estimator bias (see, e.g., [15,16]. Hence, effective estimation requires a careful trade-off between bias and variance. In practice, cross-validation remains the most widely used data-driven approach for selecting the bandwidth. As in finite-dimensional settings, the resulting optimal bandwidth is random, as it depends on the observed sample. Nevertheless, most theoretical results in the literature assume deterministic bandwidths. This consideration limits the practical relevance of the theory, since real-world applications invariably involve random, data-dependent bandwidths. To address this limitation, we study the uniform-in-bandwidth asymptotic properties of the functional ($L^{1}$)-modal regression estimator, which is constructed via a robust quantile regression approach. This strategy enhances the robustness of conditional mode estimation. The asymptotic results are established under general conditions, including assumptions on the concentration of the functional regressors, smoothness of the model, and entropy properties. Finally, the practical values of the estimator as well as the obtained asymptotic results are emphasized for constructing data-driven bandwidth selectors permitting automated implementation of the estimator.

We note that uniform consistency over the bandwidth has been extensively studied in the finite-dimensional case (cf. [17,18,19,20], for early results, and [21,22,23], for recent advances). These works cover much of the multivariate nonparametric kernel estimation literature. To the best of our knowledge, however, the present study represents the first treatment of uniform-in-bandwidth consistency of the functional ($L^{1}$)-modal regression in the context of finite or infinite-dimensional data framework.

This paper is organized as follows. In Section 2, we introduce the proposed estimation algorithms. Section 3 presents our functional framework. The necessary assumptions and the main asymptotic results are presented in Section 4. In Section 6, we discuss the impact of the asymptotic result on the selection of the smoothing parameter. The performance of the proposed estimator is evaluated on both simulated and real datasets in Section 7. Finally, Section 8 provides concluding remarks. Technical proofs are collected in Appendix A.

## 2. The Functional $L^{1}$-Modal Regression and Its Estimator

Let ${\left\{\left(U_{i},V_{i}\right)\right\}}_{i=1}^{n}$ be a sample of independent observations drawn from the joint distribution of the random pair (U,V), taking values in W×R, where W is a semi-metric space endowed with the semi-metric $S_{d}$.

Let U∈W be fixed, and let $N_{U}$ denote a neighborhood of U. We assume that a regular version of the conditional distribution function of *V* given U=U exists, and we denote it by Cdf(·∣U). In addition, we assume that this conditional distribution function is strictly increasing and admits a continuous density, denoted by Pd(v∣U), with respect to the Lebesgue measure on R.

Under these assumptions, the conditional mode at U is defined as the value that maximizes the conditional density over a given compact set *S*, that is,

$$LM\left(U\right)=\arg\max_{v\in S}Pd\left(v|U\right).$$

In this work, we adopt an alternative strategy based on the derivative of the conditional quantile function, which offers improved robustness properties. Let $t\in Cdf^{-1}\left(S|U\right)=\left[a_{U},b_{U}\right]\subset \left(0,1\right)$, and denote by α(t∣U) the conditional quantile function of order α given U=U. Motivated by arguments similar to those of [24] in the linear framework, we assume that α(t∣U) is continuously differentiable (i.e., of class $C^{1}$) with respect to α, and that

$$Cdf\left(\alpha (t|U)|U\right)>0.$$

Under these conditions, the derivative of the conditional quantile function satisfies

$$\alpha^{\prime }\left(t|U\right)=\frac{\partial \alpha (t|U)}{\partial t}=\frac{1}{Cdf\left(\alpha (t|U)|U\right)}.$$

Consequently, the $L^{1}$ modal regression can equivalently be characterized as

$$LM\left(U\right)=\alpha \left(t^{*}|U\right),\;\mathrm{where}\;t^{*}=\arg\min_{t\in [a_{U},b_{U}]}\alpha^{\prime }\left(t|U\right).$$

Consequently, a natural estimator of the conditional mode is defined by

(1) $$\hat{LM}\left(U\right)=\hat{\alpha }\left(\hat{t^{*}}|U\right),$$

where

$$\hat{t^{*}}=\arg\min_{\alpha \in [a_{U},b_{U}]}\hat{\alpha^{\prime }}\left(t|U\right),$$

and $\hat{\alpha }$ and $\hat{\alpha^{\prime }}$ denote estimators of the conditional quantile function and its derivative, respectively. For numerical implementation, the derivative is approximated by the symmetric difference quotient

$$\hat{\alpha^{\prime }}\left(t|U\right)=\frac{\hat{\alpha }\left(t+d_{n}|U\right)-\hat{\alpha }\left(t-d_{n}|U\right)}{2d_{n}},$$

where $d_{n}>0$ is a sequence of smoothing parameters playing a role similar to a bandwidth in kernel-based methods.

To obtain a robust estimator of the conditional quantile, recall that the *t*-th conditional quantile α(t∣U) is the unique minimizer of

(2) $$\min_{s\in R}E\;\left[\varrho_{\alpha }\left(V,s\right)|U=U\right],$$

where $\varrho_{\alpha }\left(V,s\right)=\left(2\alpha -1\right)\left(V-s\right)+\left|V-s\right|$ denotes the check loss function. Accordingly, the estimator of the conditional quantile is defined by

(3) $$\hat{\alpha }\left(t|U\right)=\arg\min_{s\in R}\hat{\Psi }\left(s,\alpha |U\right),$$

with

$$\hat{\Psi }\left(s,\alpha |U\right)=\frac{\sum_{i=1}^{n}W\;\left(d_{n}^{-1}S_{d}\left(U,U_{i}\right)\right)\varrho_{\alpha }\left(V_{i}-s\right)}{\sum_{i=1}^{n}W\;\left(d_{n}^{-1}S_{d}\left(U,U_{i}\right)\right)},\;s\in R,$$

where W is a kernel function and $\left(d_{n}\right)$ is a sequence of positive smoothing parameters such that $d_{n}\to 0$ as n→∞.

The existence and uniqueness of LM(U) follow from the continuity and strict monotonicity of F(·∣U). While the estimator $\hat{LM}\left(U\right)$ is not necessary unique. Thus, throughout this paper, any value associated with a minimizer in (3) is regarded as an admissible estimator.

## 3. Entropy and High-Dimensional Data Framework

Recall that our main aim is to investigate the asymptotic behavior of $\hat{LM}\left(U\right)$. For this aim, we consider a measurable kernel function W(·) supported on the interval (0,1/2), and define the following classes of functions:

$$W=\left\{u\mapsto W\left(\frac{S_{d}\left(U,u\right)}{\gamma }\right),\;\gamma >0\right\},$$

Throughout this paper, $N(\epsilon ,C,d_{Q})$ denotes the minimal number of open balls of radius ϵ>0 required to cover a set of functions C with respect to the $L_{2}\left(Q\right)$ metric. For any mapping ψ:C→R, we write

$${∥\psi ∥}_{C}=\underset{g\in C}{\sup}\left|\psi \left(g\right)\right|.$$

***Functionality Assumption.*** The High-Dimensional Data framework are characterized by:

(4) $$\begin{array}{cc} \; & \mathrm{For}\;\mathrm{all}\;r>0,\;I\;P\left(X\in B\left(U,r\right)\right)=:P_{U}\left(r\right)>0, \end{array}$$

(5) $$\begin{array}{cc} \; & \mathrm{and}\;\mathrm{for}\;\mathrm{all}\;s\in \left(0,1\right),\;\lim_{r\to 0}\frac{P_{U}\left(sr\right)}{P_{U}\left(r\right)}=Q_{U}\left(s\right). \end{array}$$

These conditions are standard in the asymptotic theory of nonparametric functional statistics. In particular, (4) is a fundamental requirement (see [5], for a detailed discussion), and (5) appears frequently in related studies (cf. [25]), which also provide examples of small ball probabilities satisfying this assumption.

***Measurability Assumption.***

(6) Warepointwisemeasurableclasses.

Recall that, a class C is pointwise measurable if there exists a countable subclass $C_{0}\subset C$ such that for each g∈C, there exists a sequence ${\left(g_{m}\right)}_{m\in N}\subset C_{0}$ with $|g_{m}\left(z\right)-g\left(z\right)|\to 0$ for all *z*. This mild measurability condition allows us to present results in the standard probabilistic framework, avoiding the more complex notions of outer probability or outer expectation (cf. [17]).

***Entropy Assumption.***

(7) $$\begin{array}{cc} \; & J\left(1,W\right)=\underset{Q}{\sup}\int_{0}^{1}\sqrt{1+\log N(\epsilon ∥F{∥}_{Q,2},W,d_{Q})}\;d\epsilon <\infty , \end{array}$$

where *F* is the envelope function of W, and $d_{Q}$ (resp. ${∥\cdot ∥}_{Q,2}$) denotes the $L_{2}\left(Q\right)$ metric (resp. norm). The supremum is taken over all probability measures *Q* on the functional space F. These entropy conditions are closely connected to uniform asymptotic results, providing control over the uniformity in bandwidth and characterizing Donsker-type function classes (cf. [26]). They are more general than the classical VC-class condition (cf. [26]).

Overall, these three assumptions are sufficiently mild and encompass all relevant aspects of our uniform consistency results, including the topological structure of the functional covariates, the probability measure on the functional space, the measurability of function classes, and the uniformity ensured by the entropy condition.

## 4. Main Result

Now, to establish the UBC of $\hat{LM}$, over the bandwidth parameter $d_{n}\in \left(A_{n},B_{n}\right)$ we consider following assumptions:

ML1 The functions α(·|U) is of class $C^{3}\left(\left[a_{U},b_{U}\right]\right)$ and Cdf(·|U) such that the following Lipschitz’s condition
  $$\mathrm{for}\;\mathrm{all}\;\left(U_{1},U_{2}\right)\in N_{U},\;\;\left|F\left(t|U_{1}\right)-F\left(t|U_{2}\right)\right|\leq Cd^{b}\left(U_{1},U_{2}\right)\;\mathrm{for}\;\mathrm{some}\;b>0,$$
  where $N_{U}$ denotes a neighborhood of *x*.
ML2 The kernel W is supported within (0,1/2) and has a continuous first derivative on (0,1/2), such that:
  (8) $${C1}_{\left(0,1/2\right)}\left(\cdot \right)\leq W\left(\cdot \right)\leq {C1}_{\left(0,1/2\right)}^{\prime }\left(\cdot \right)\;\;\mathrm{and}\;\;W\left(1/2\right)-\int_{0}^{1/2}W^{\prime }\left(s\right)Q_{U}\left(s\right)ds>0$$
ML3 The sequence $\left(A_{n}\right)$ verifies:
  (9) $$\frac{\log n}{n\min(A_{n},P_{U}\left(A_{n}\right))}\to 0.$$

Under these assumptions, we obtain the following uniform consistency result:

**Theorem** **1.** *Under (ML1)–(ML3), we have*

$$\underset{A_{n}\leq d_{n}\leq B_{n}}{\sup}\left|\hat{LM}\left(U\right)-LM\left(U\right)\right|=O\left(B_{n}^{\beta }\right)+O_{a.co.}{\left(\frac{\log n}{nP_{U}\left(A_{n}\right)}\right)}^{1/4}.$$

*where β=min(b,1)/2.*

## 5. Proof of Theorem 1

**Proof of Theorem** **1.** The proof of Theorem 1 is based on standard analytical arguments. We begin by decomposing the estimation error of the conditional mode:

(10) $$\begin{array}{cc} \hat{LM}\left(U\right)-LM\left(U\right)\; & =\hat{\alpha }\left({\hat{t}}^{*}|U\right)-\alpha \left(t^{*}|U\right)\\ \; & =\underset{\mathrm{quantile}\;\mathrm{estimation}\;\mathrm{error}}{\underset{︸}{\hat{\alpha }\left({\hat{t}}^{*}|U\right)-\alpha \left({\hat{t}}^{*}|U\right)}}+\underset{\mathrm{error}\;\mathrm{due}\;\mathrm{to}\;t^{*}}{\underset{︸}{\alpha \left({\hat{t}}^{*}|U\right)-\alpha \left(t^{*}|U\right)}}\\ \; & \leq \underset{t\in [a_{U},b_{U}]}{\sup}\left|\hat{\alpha }\left(t|U\right)-\alpha \left(t|U\right)\right|+\left|\alpha \left({\hat{t}}^{*}|U\right)-\alpha \left(t^{*}|U\right)\right|. \end{array}$$

The second term can be handled using a Taylor expansion:

(11) $$\alpha \left(\hat{t^{*}}|U\right)-\alpha \left(t^{*}|U\right)=\left(\hat{t^{*}}-t^{*}\right)\;\alpha^{\prime }\left(t^{{*}^{*}}|U\right),$$

for some $t^{{*}^{*}}$ between $\hat{t^{*}}$ and $t^{*}$.

Since $t^{*}$ is the minimizer of $\alpha^{\prime }\left(\cdot |U\right)$, we can further write

(12) $$\alpha^{\prime }\left(\hat{t^{*}}|U\right)-\alpha^{\prime }\left(t^{*}|U\right)={\left(\hat{t^{*}}-t^{*}\right)}^{2}\;\alpha^{\prime \prime \prime }\left(t^{{*}^{**}}|U\right),$$

for some $t^{{*}^{**}}$ between $\hat{t^{*}}$ and $t^{*}$.

Moreover, we can decompose the derivative estimation error as

(13) $$\begin{array}{cc} \alpha^{\prime }\left(\hat{t^{*}}|U\right)-\alpha^{\prime }\left(t^{*}|U\right)\; & =\left(\alpha^{\prime }\left(\hat{t^{*}}|U\right)-\hat{\alpha^{\prime }}\left(\hat{t^{*}}|U\right)\right)+\left(\hat{\alpha^{\prime }}\left(\hat{t^{*}}|U\right)-\alpha^{\prime }\left(t^{*}|U\right)\right)\\ \; & \leq |\alpha^{\prime }\left(\hat{t^{*}}|U\right)-\hat{\alpha^{\prime }}\left(\hat{t^{*}}|U\right)|+|\min_{t}\hat{\alpha^{\prime }}\left(t|U\right)-\min_{t}\alpha^{\prime }\left(t|U\right)|\\ \; & \leq 2\underset{t\in [a_{U},b_{U}]}{\sup}\left|\hat{\alpha^{\prime }}\left(t|U\right)-\alpha^{\prime }\left(t|U\right)\right|. \end{array}$$

Combining (10)–(13), we obtain

$$|\hat{LM}\left(U\right)-LM\left(U\right)|\leq C\left(\underset{\alpha \in [a_{U},b_{U}]}{\sup}\left|\hat{\alpha }\left(t|U\right)-\alpha \left(t|U\right)\right|+\sqrt{\underset{\alpha \in [a_{U},b_{U}]}{\sup}\left|\hat{\alpha^{\prime }}\left(t|U\right)-\alpha^{\prime }\left(t|U\right)\right|}\right).$$

Next, using the definition of $\hat{\alpha^{\prime }}\left(t|U\right)$, we have for *n* sufficiently large,

$$\begin{array}{cc} \hat{\alpha^{\prime }}\left(t|U\right)-\alpha^{\prime }\left(t|U\right) & =\frac{\hat{\alpha }\left(t+d_{n}|U\right)-\alpha \left(t+d_{n}|U\right)+\alpha \left(t-d_{n}|U\right)-\hat{\alpha }\left(t-d_{n}|U\right)}{2d_{n}}\\ & \;+\frac{\alpha \left(t+d_{n}|U\right)-\alpha \left(t|U\right)+\alpha \left(t|U\right)-\alpha \left(t-d_{n}|U\right)-2d_{n}\alpha^{\prime }\left(t|U\right)}{2d_{n}}\\ & \leq Cd_{n}^{-1}\underset{\alpha \in (a_{U}-d_{n},b_{U}+d_{n})}{\sup}\left|\hat{\alpha }\left(t|U\right)-\alpha \left(t|U\right)\right|+O\left(d_{n}\right). \end{array}$$

Hence, it is sufficient to control the quantity

$$\underset{A_{n}\leq d_{n}\leq B_{n}}{\sup}\underset{t\in [0,1]}{\sup}\left|\hat{\alpha }\left(t|U\right)-\alpha \left(t|U\right)\right|.$$

It has been shown in [14] that the conditional quantile estimator admits a Bahadur-type representation that can be expressed explicitly as

$$\hat{\alpha }\left(t|U\right)-\alpha \left(t|U\right)=\frac{1}{Pd(t_{t}\left(U\right)|U)}C_{n}\left(t,d_{n}\right)+$$

$$O\;\left(\underset{|\delta |\leq M}{\sup}\left|D_{n}\left(t,\delta ,d_{n}\right)+Pd\left(t_{t}\left(U\right)|U\right)\delta -C_{n}\left(t,d_{n}\right)\right|\right).$$

Here,

$$D_{n}\left(t,\delta ,d_{n}\right)=\frac{1}{nP_{U}\left(d_{n}\right)}\sum_{i=1}^{n}\left(t-1_{\left\{V_{i}\leq \left(\delta +\alpha \left(t|U\right)\right)\right\}}\right)W\;\left(d_{n}^{-1}S_{d}\left(U,U_{i}\right)\right)$$

$$\mathrm{and}\;C_{n}\left(t,d_{n}\right)=D_{n}\left(t,0,d_{n}\right).$$

Therefore, the desired result follows directly from the two lemmas stated below. □

**Lemma** **1.** *Under the assumptions of Theorem 1, we have*

$$\underset{A_{n}\leq d_{n}\leq B_{n}}{\sup}\underset{t\in [0,1]}{\sup}\left|C_{n}\left(t,d_{n}\right)\right|=O\left(B_{n}^{\beta }\right)+O_{a.co.}\;\left(\sqrt{\frac{\log n}{n\;P_{U}\left(A_{n}\right)}}\right).$$

**Lemma** **2.** *Under the assumptions of Theorem 1, we obtain*

$$\underset{A_{n}\leq d_{n}\leq B_{n}}{\sup}\underset{t\in [0,1]}{\sup}\underset{|\delta |\leq M}{\sup}\left|D_{n}\left(t,\delta ,d_{n}\right)+Pd\left(t_{t}\left(U\right)\right)\;\delta -C_{n}\left(t,d_{n}\right)\right|=O\left(B_{n}^{\beta }\right)$$

$$+O_{a.co.}\;\left(\sqrt{\frac{\log n}{n\;P_{U}\left(A_{n}\right)}}\right).$$

## 6. Application: Automatic Data-Driven Bandwidth Selection for Modal Regression

In nonparametric regression analysis, the selection of an appropriate bandwidth is challenging issues. This choice should be driven by the data itself, which means that the bandwidths employed are not fixed constants but random variables that depend on the observed sample. In this context, our theoretical results play a crucial role, as they provide a mathematical support that take into account for the randomness of data-driven bandwidth selection. Typically, consider the estimator $\hat{ML}$ with random bandwidth $\tilde{d_{n}}$ taking values in the interval $\left(A_{n},B_{n}\right)$ where $A_{n}$ and $B_{n}$ are defined by (9). Then, if we denote by $\tilde{ML}$ the same kernel estimator as $\hat{ML}$ obtained by pluging the random bandwidth $\tilde{d_{n}}$, then we determine, as a direct consequence of Theorem 1, that:

(14) $$|\tilde{LM}\left(U\right)-LM\left(U\right)|=O\left(B_{n}^{\beta }\right)+O_{a.co.}{\left(\frac{\log n}{nP_{U}\left(A_{n}\right)}\right)}^{1/4}.$$

In practice, we need to use data-driven bandwidths, i.e., choosing bandwidths in function of the statistical sample:

$$\tilde{d_{n}}=\tilde{d}\left(U_{1},V_{1},\ldots ,U_{n},V_{n}\right).$$

Therefore, consistency results, with rates, can be obtained for any kind of automatic data-driven bandwidth selector directly from (14). The most popular technique is the cross-validation method which is obtained by the minimization of the following rules:

- The mean least square error is defined by
  (15) $${\tilde{d}}_{LSCV}=\arg\min_{d\in (A_{n},B_{n})}CV\left(d_{n}\right),$$
  where
  $$CV\left(d_{n}\right)=\sum_{i=1}^{n}{\left(V_{i}-\hat{V_{i}}\right)}^{2}$$
- The qunatile cross-validation rule based on
  (16) $${\tilde{d}}_{QUCV}=\arg\min_{d\in (A_{n},B_{n})}CV\left(d_{n}\right)$$
  where
  $$CV\left(d_{n}\right)=\sum_{i=1}^{n}L_{p}\left(V_{i}-\hat{V_{i}}\right)$$

in both case $\hat{V_{i}}$ is the statistical prediction of $V_{i}$ obtained by using the estimator $\hat{ML}$ based on the leave-out-one-curve sample. Then, we have the following result.

**Corollary** **1.** *Under the conditions of Theorem 1, if ${\tilde{ML}}_{CV}$ is the estimator $\hat{ML}$ constructed with the cross-validated bandwidth ${\tilde{d}}_{LSCV}$ (resp. ${\tilde{d}}_{QUCV}$), then we have:*

$$|{\tilde{LM}}_{CV}\left(U\right)-LM\left(U\right)|=O\left(B_{n}^{\beta }\right)+O_{a.co.}{\left(\frac{\log n}{nP_{U}\left(A_{n}\right)}\right)}^{1/4}.$$

This result has a significance impact in practice, as it provides theoretical justification for the widely used data-driven cross-validation procedure. Therefore, it makes the application of functional $L^{1}$ modal regression more easy and more attractive.

## 7. Data-Driven Analysis

### 7.1. A Simulated Data Case

In this section, we present simulated examples to illustrate the effectiveness of the theoretical results established in this contribution. Specifically, our objectives are:

1. To demonstrate that the proposed estimator is easy for practical implementation.
2. To highlight the advantages of employing the obtained results to the bandwidth selection.

Specifically, we compare the two bandwidth selection methods introduced in the previous section. In addition, we examine their performance under both global and local smoothing parameter selection strategies. For this aim, we consider challenging functional curves that incorporate heteroscedasticity in our functional nonparametric structure model. Such strategy allows to emphasize the practical benefits and robustness of our approach. Indeed, we generate the functional data as follows:

(17) $$V_{i}=r\left(U_{i}\right)+\sigma_{1,\varepsilon }\varepsilon_{i},\;i\in S_{1}=\left\{1,\ldots ,200\right\},$$

$$V_{i}=r\left(U_{i}\right)+\sigma_{2,\varepsilon }\varepsilon_{i},\;i\in S_{2}=\left\{201,\ldots ,400\right\},$$

where the random errors $\varepsilon_{i}$ are independently drawn from a N(0,1) distribution. The noise levels $\sigma_{1,\varepsilon }$ and $\sigma_{2,\varepsilon }$ are calibrated through different values of the signal-to-noise ratio (SNR_*k*_), specifically 5%, 10%, 20%, 30%, and 50%. For k=1,2, the signal-to-noise ratio is defined as

$${\mathrm{SNR}}_{k}=\frac{\sigma_{W,\varepsilon }^{2}}{\frac{1}{200}\sum_{i\in S_{k}}{\left(r\left(U_{i}\right)-{\bar{r(U)}}_{S_{k}}\right)}^{2}}.$$

The functional covariates $U_{i}$, i=1,…,400, are generated according to

$$U_{i}\left(t\right)=a_{i}\cos\;\left(4(b_{i}-t)\right)+b_{i}+c\eta_{i,t},\;t\in \left[0,1\right].$$

**Figure 1:**
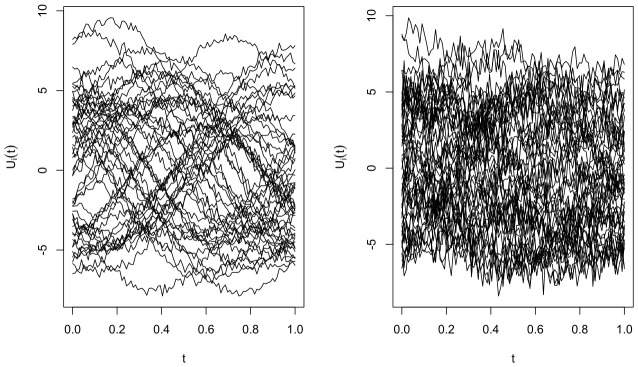
A sample of two types of curves (low and high variability).

Here, $b_{i}∼N\left(0,2\right)$ and $a_{i}∼N\left(-5,0.5\right)$. For the first 200 observations $\eta_{i}∼N\left(0,0.05\right)$, while the remaining 200 are sampled by $\eta_{i}∼N\left(0,1\right)$ The two types functional trajectories $U_{i}$ are discretized on a common grid of 100 equally spaced points over the interval [0,1] (see Figure 1).

On the other hand, the scalar response variable $V_{i}$, defined in (17), is generated through the regression operator

$$r\left(U\right)=3*\int_{0}^{1}\frac{1}{1+|U(t)|}\;dt.$$

It is worth emphasizing that this model specification (17) permits to introduce the heteroscedastic structure which provide a particularly suitable framework for assessing and comparing the predictive performance of the proposed estimator under local and global bandwidth selection strategies.

For practical implementation, the parameters ${\tilde{d}}_{LSCV}$ (respectively, ${\tilde{d}}_{QUCV}$) are selected from a finite grid of candidate values. More precisely, under the local selection rule, the optimization is carried out over a subset $H_{n}\left(U\right)$ associated with the location point U. This subset consists of the positive real numbers *a* such that the ball centered at U with radius *a* contains exactly *k* observations among the $\left(U_{i}\right)$. In contrast, under the global strategy, the set $H_{n}$ is defined independently of the location point U. Specifically, it is constructed using the empirical quantile of order *q* computed from the vector of all pairwise distances between the curves. Finally, the predictive performance of both selection procedures is assessed through the computation of the relative mean squared prediction error (*MSE*) defined as follows:

$$MSE_{CV}=\frac{1}{n}\sum_{i=1}^{n}{\left(V_{i}-{\tilde{ML}}^{CV}\left(U_{i}\right)\right)}^{2}$$

where ${\hat{ML}}^{CV}$ means the $L^{1}$ estimator of the modal regression computed by the local or global versions of the rules described in the previous section. We point out that, we employ a quadratic kernel function. Given the structural characteristics of the generated curves, we adopt a semi-metric derived from functional principal component analysis (see [5], considering several values of the truncation dimension *p*. We report only the result of p=3. The corresponding results are reported in Table 1.

Table 1 reports the *MSE* values of the two selectors for different levels of the signal-to-noise ratio (SNR_*k*_). The results clearly indicate that the QUCV rule outperforms the LSCV criterion. Specifically, the *MSE* values range between (0.16,0.40) for QUCV, compared with (0.27,0.63) for the LSCV rule. Furthermore, the local bandwidth selection method consistently performs better than the global bandwidth selection approach. This conclusion is again supported by the observed *MSE* values, which show that the global strategy yields comparatively poor performance. It is also worth noting that predictive accuracy depends on the underlying data characteristics, and that the *RMSE* increases as the signal-to-noise ratio becomes larger. However, the magnitude of this increase remains moderate, highlighting the robustness of the $L^{1}$-mode regression estimator.

In the second illustration, we compare the performance of the $L^{1}$-modal regression estimator $\hat{ML}$ with that of the $L^{1}$-median regression estimator, denoted by

$$\hat{ME}=\arg\min_{s}\frac{\sum_{i=1}^{n}W\;\left(d_{n}^{-1}S_{d}\left(U,U_{i}\right)\right)\left|V_{i}-s\right|}{\sum_{i=1}^{n}W\;\left(d_{n}^{-1}S_{d}\left(U,U_{i}\right)\right)},$$

**Figure 2:**
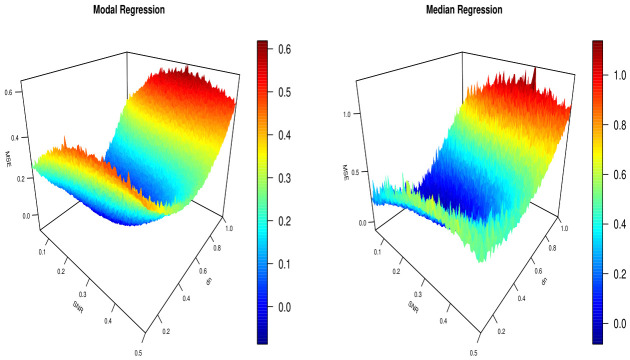
Comparison of MSE errors between modal and median regression.

To this aim, the sample is randomly divided into a learning set of 300 observations and a test set of 100 observations. Each subset contains an equal number of observations from $S_{1}$ and $S_{2}$. We calculate both predictors by adopting the same kernel and metric as in the first illustration. To evaluate the robustness of the predictors $\hat{ML}$ and $\hat{ME}$, we compute the mean square error as function of the bandwidth values $d_{n}\in H_{n}$ and SNR values

$$MSE\left(d_{n},SNR\right)=\frac{1}{100}\sum_{i=1}^{100}{\left(V_{i}-\tilde{\theta }\left(U_{i}\right)\right)}^{2}$$

where $\tilde{\theta }\left(\cdot \right)$ means $\hat{ML}$ and $\hat{ME}$. Figure 2 displays the multi-variable function MSE for different levels of SNR and various bandwidth values. The results clearly show the robustness of both models with substantial superiority of the model predictor $\hat{ML}$ in term of accuracy and robustness. The latter provides a small variation of the error. We point out that the parabolic form of the error with respect to the $d_{n}$-variable confirms the role of the smoothing parameter and the importance of our contribution to providing an automatic approach to select this parameter.

### 7.2. Real Data Application

This section is devoted to apply the $L^{1}$-modal regression model as a functional predictor in real data. We emphasize the accuracy and robustness of the proposed approach by conducting a comparative evaluation against several competing regression predictors. In particular, our empirical analysis focuses on estimating the starch content of potatoes from near-infrared (NIR) spectral curves. Recall that the accurate prediction of starch concentration in potatoes is of considerable scientific and industrial importance. Starch is a principal nutritional component, it has beneficial effects on digestive health and potential protective roles against certain pathological conditions. From an agronomic and food-processing perspective, reliable quantification of starch content is essential for quality control, product classification, and optimization of industrial transformation processes (such as, chips, fries, and starch-derived products). Consequently, developing accurate and robust predictive tools for starch assessment is both economically and nutritionally significant.

In this contribution, we use near-infrared (NIR) spectrometry to characterize and model the relationship between spectral signatures and starch concentration in potatoes. The functional NIR dataset analyzed in this study is available at http://www.models.life.ku.dk (accessed on 3 March 2026). It is widely recognized that nonparametric functional statistical methods offer a flexible and powerful approach for the analysis of NIR spectroscopy data. By representing each spectrum as a functional observation rather than as a finite-dimensional vector, we can exploit the smooth variation across wavelengths and preserve the functional structure of the curves. This functional treatment improves the predictive performance and enhances the interpretability and stability of the resulting regression models.

**Figure 3:**
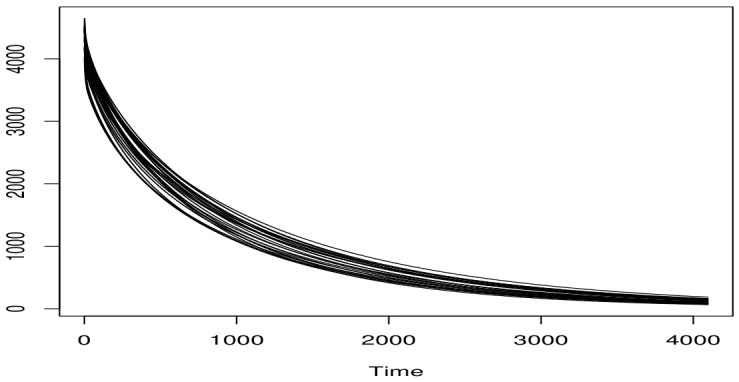
The spectral curves.

The corresponding spectral curves are displayed in Figure 3. Additional technical details regarding the experimental design and data collection procedure can be found in [27].

For the prediction task, we compare the performance of three predictors that are $\hat{LM}$ and $\hat{ME}$:

$$\begin{array}{c} \hat{MO}\left(U\right)=\arg\max_{v}\frac{\sum_{i=1}^{n}W\;\left(d_{n}^{-1}S_{d}\left(U,U_{i}\right)\right)W\;\left(d_{n}^{-1}\left(V_{i}-v\right)\right)}{\sum_{i=1}^{n}W\;\left(d_{n}^{-1}S_{d}\left(U,U_{i}\right)\right)}. \end{array}$$

To guarantee a fair and consistent comparison, identical computational settings were adopted for all three estimators. In particular, we employed the same PCA-metric and applied the bandwidth selection rule defined in Equation (15) for each method. This unified framework ensures that any observed differences in predictive performance are attributable to the properties of the estimators.

**Figure 4:**
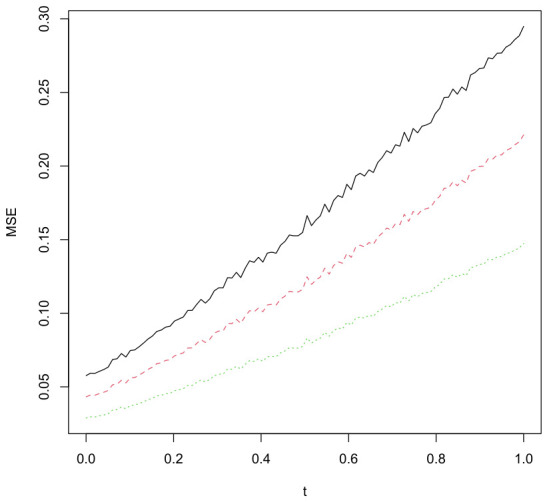
Comparison of prediction errors for three predictor $\hat{LM}$ (green) and $\hat{ME}$ (red) and $\hat{MO}$ (black).

Next, to examine the robustness of the competing estimators, we introduced contamination into the dataset by multiplying a subset of the observations by a factor of 10. The effect of this perturbation was evaluated by monitoring changes in the Mean Absolute Error (MAE). More precisely, for a given contamination level t∈[0,1], we multiplied [tn] observations (where [.] denotes the integer part) and computed the variability of the prediction error function using

$$\mathrm{MSE}\left(t\right)=\frac{1}{26}\sum_{k=1}^{26}{\left(V_{k}-\dot{Pred}\left(U_{k}\right)\right)}^{2},$$

where $\dot{Pred}$ represents any one of the three estimators $\hat{LM}$ and $\hat{ME}$ and $\hat{MO}$ The resulting prediction errors, as a function of the contamination proportion, are reported in Figure 4. It provides a clear illustration of the relative stability and resistance of each method to outlying observations.

The results show that, although the three predictors exhibit satisfactory performance in the absence of contamination (t=0), the $L^{1}$-based approaches clearly outperform their competitors once perturbations are introduced. In particular, the $L^{1}$-modal regression estimator ($\hat{ML}$) demonstrates remarkable stability, as reflected by the lower variability of its MsE under increasing contamination levels. This robustness is quantitatively supported by the range of the error function. The MAE associated with the $\hat{ML}$ regression varies in the interval ([0.02, 0.15]), whereas for the predictor $\hat{ME}$ the corresponding ranges are ([0.02, 0.26]) and ([0.05, 0.34]) for the standard nonparametric modal regression $\hat{MO}$. This statement incorporates the theoretical expectations: estimators based on the M-estimation of the quantiles is less sensitive to extreme residuals limiting the impact of large deviations and outliers.

## 8. Conclusions and Prospects

The present study develops a robust framework for functional modal regression based on the M-estimation of the qunatile regression. Under this structure, we construct a new modal regression estimator and we establish its asymptotic properties, including complete consistency. The functional nature of the explanatory variable is handled through the small ball probability function, which captures the topological structure of the underlying probability measure and plays a central role in controlling the stochastic behavior of the estimator. By incorporating the $L^{1}$-criterion, the proposed method enhances robustness, limiting the influence of extreme deviations and yielding more stable inference. Clearly, estimating the conditional mode is of particular importance in many practical contexts, as it provides a meaningful measure of central tendency in asymmetric or heavy-tailed distributions, where standard prediction based on conditional mean is inadequate. Our theoretical contribution of this work is the establishment of uniform consistency with respect to the bandwidth parameter. Demonstrating uniform convergence is essential, as it provides the mathematical foundation required to justify a wide range of smoothing parameter selection procedures. In particular, this result ensures the consistency of data-driven bandwidth choices such as cross-validation or plug-in methods—by guaranteeing that the estimator remains stable uniformly over admissible bandwidth sequences. Hence, the theoretical analysis not only validates the estimator itself but also offers mathematical support for practical implementation strategies. The asymptotic results are derived under general and flexible assumptions, making them applicable to a broad class of functional data and continuous-time processes. Empirical investigations confirm that the estimator is straightforward to implement and that several reliable techniques are available for selecting the principal smoothing parameters. The numerical results illustrate the strong predictive performance and robustness of the proposed procedure when compared with alternative regression approaches.

This contribution opens several promising questions for future research. A natural extension would be the development of a local linear version of the robust modal regression estimator, which could reduce boundary bias and improve convergence rates. Another important direction is the derivation of asymptotic normality, a necessary step toward constructing confidence intervals and conducting formal hypothesis testing. Additional extensions may include linear or partially linear functional regression structures, as well as adaptations to more complex settings such as censored observations or functional time series. Addressing these challenges will require new theoretical tools and refined probabilistic techniques, offering fertile ground for further methodological advances.

## Figures and Tables

**Table 1:** Mean square errors according to various situations.

	Functional Variability	SNR				
	Low Variability	**5%**	**10%**	**20%**	**30%**	**50%**
rmse_*LSCV*_*LOC*__		0.2773	0.3159	0.3696	0.4013	0.4308
rmse_*LSCV*_*GLOB*__		0.4127	0.4861	0.5178	0.5716	0.6153
rmse_*QUCV*_*LOC*__		0.1678	0.1865	0.17243	0.2091	0.21456
rmse_*QUCV*_*GLOB*__		0.1965	0.20254	0.21267	0.2233	0.2417
	High variability					
rmse_*LSCV*_*LOC*__		0.3451	0.3791	0.4016	0.4161	0.4611
rmse_*LSCV*_*GLOB*__		0.3842	0.51472	0.5271	0.5614	0.6315
rmse_*QUCV*_*LOC*__		0.26148	0.2814	0.29124	0.3053	0.31617
rmse_*QUCV*_*GLOB*__		0.3115	0.3215	0.3651	0.3913	0.4018

## Data Availability

The data used in this study are available through the link https://uk-air.defra.gov.uk/data/ (accessed on 3 March 2026).

## Appendix A

For brevity, we establish only the second lemma since the proof of the first lemma follows along very similar lines. For convenience, let us denote, for every i=1,…,n,

$$W_{i}=W\;\left(d_{n}^{-1}S_{d}\left(U,U_{i}\right)\right),$$

and define

$$V_{n}\left(t,\delta ,d_{n}\right)=D_{n}\left(t,\delta ,d_{n}\right)-C_{n}\left(t,d_{n}\right).$$

Let us precise that the proof is based on the following inequality.

**Lemma** **A1** (cf. Theorem 3.1 in [17])**.** *Let $Z_{1},Z_{2},\ldots ,Z_{n}$ be independent and identically distributed F-valued random variables and consider C a pointwise measurable class of functions g:F→R with envelope function F. Assume that for some H>0,*

$$I\;E\left[F^{p}\left(Z\right)\right]\leq \frac{p!}{2}\sigma^{2}H^{p-2}\;\mathrm{where}\;\sigma^{2}\geq I\;E\left[F^{2}\left(Z\right)\right].$$

*Then, for $\beta_{n}=I\;E[∥\sqrt{n}\alpha_{n}\left(g\right){∥}_{C}]$, we have for any t>0*

$$I\;P\left\{\max_{1\leq k\leq n}{∥\alpha_{n}\left(g\right)∥}_{C}\geq \beta_{n}+t\right\}\leq \exp\left(-\frac{t^{2}}{2n\sigma^{2}+2tH}\right)$$

**Proof** **of Lemma 2.** The proof based on the compactness of the intervals [0,1] and [−M,M]. In particular, we may construct finite coverings such that

$$\left[0,1\right]\subset \overset{d_{n}}{\underset{k=1}{⋃}}\left[t_{k}-l_{n},t_{k}+l_{n}\right],\;t_{k}\in \left[0,1\right],$$

and

$$\left[-M,M\right]\subset \overset{d_{n}}{\underset{j=1}{⋃}}\left[\delta_{j}-l_{n},\delta_{j}+l_{n}\right],\;\delta_{j}\in \left[-M,M\right],$$

where $l_{n}=d_{n}^{-1}=1/\sqrt{n}$. For any δ∈[−M,M], define

$$j\left(\delta \right)=\arg\min_{j}\left|\delta -\delta_{j}\right|,$$

and for any t∈[0,1], define

$$k\left(t\right)=\arg\min_{k}\left|t-t_{k}\right|.$$

We then analyze the quantity as a function of δ and *t*. Consequently,

$$\begin{array}{c} \underset{|\delta |\leq M}{\sup}\underset{t\in [0,1]}{\sup}\left|V_{n}\left(t,\delta ,d_{n}\right)-I\;E\left[V_{n}\left(t,\delta ,d_{n}\right)\right]\right|\\ \;\;\;\leq \underset{|\delta |\leq M}{\sup}\underset{t\in [0,1]}{\sup}\left|V_{n}\left(t,\delta ,d_{n}\right)-V_{n}\left(t,\delta_{j(\delta )},d_{n}\right)\right|\\ \;\;\;\;\;\;+\underset{|\delta |\leq M}{\sup}\underset{t\in [0,1]}{\sup}\left|V_{n}\left(t,\delta_{j(\delta )},d_{n}\right)-V_{n}\left(t_{k(t)},\delta_{j(\delta )},d_{n}\right)\right|\\ \;\;\;\;+\underset{|\delta |\leq M}{\sup}\underset{t\in [0,1]}{\sup}\left|V_{n}\left(t_{k(t)},\delta_{j(\delta )},d_{n}\right)-I\;E\left[V_{n}\left(t_{k(t)},\delta_{j(\delta )},d_{n}\right)\right]\right|\\ \;\;\;\;\;\;\;+\underset{|\delta |\leq M}{\sup}\underset{t\in [0,1]}{\sup}\left|I\;E\left[V_{n}\left(t_{k(t)},\delta_{j(\delta )},d_{n}\right)\right]-I\;E\left[V_{n}\left(t_{k(t)},\delta ,d_{n}\right)\right]\right|\\ \;\;\;+\underset{|\delta |\leq M}{\sup}\underset{t\in [0,1]}{\sup}\left|I\;E\left[V_{n}\left(t_{k(t)},\delta ,d_{n}\right)\right]-I\;E\left[V_{n}\left(t,\delta ,d_{n}\right)\right]\right|. \end{array}$$

To handle the third term, observe that

$$|V_{n}\left(t_{k},\delta_{j},d_{n}\right)-I\;E\left[V_{n}\left(t_{k},\delta_{j},d_{n}\right)\right]|\;=\frac{1}{n\;P_{U}\left(d_{n}\right)}\sum_{i=1}^{n}\Gamma_{i}\left(W\right),$$

where

$$\Gamma_{i}\left(W\right)=\left(1_{V_{i}\leq \alpha \left(t_{k}|U\right)}-1_{V_{i}\leq \delta_{j}+\alpha \left(t_{k}|U\right)}\right)W_{i}$$

$$\;-I\;E\;\left[\left(1_{V_{i}\leq \alpha \left(t_{k}|U\right)}-1_{V_{i}\leq \delta_{j}+\alpha \left(t_{k}|U\right)}\right)W_{i}\right].$$

It is therefore sufficient to prove the existence of $\eta_{0}^{\prime }>0$ such that, for some constant $B_{0}>0$,

$$\sum_{n}I\;P\;\left\{\underset{A_{n}\leq d_{n}\leq B_{0}}{\sup}\underset{|\delta |\leq M}{\sup}\underset{t\in [0,1]}{\sup}\sqrt{\frac{nP_{U}\left(d_{n}\right)}{\log n}}\left|V_{n}\left(t_{k},\delta_{j},d_{n}\right)-I\;E\left[V_{n}\left(t_{k},\delta_{j},d_{n}\right)\right]\right|\geq \eta_{0}^{\prime }\right\}<\infty .$$

To establish this result, we follow arguments similar to those developed by [17], which based on Bernstein’s inequality for empirical processes (see Lemma A1). For this purpose, define

$$d_{n,l}=2^{l}d_{n},\;L\left(n\right)=\max\left\{l:\;d_{n,l}\leq 2B_{0}\right\},$$

and introduce the empirical process

$$\vartheta_{n}\left(W\right)=\frac{1}{\sqrt{n}}\sum_{i=1}^{n}\left(\Gamma_{i}\left(W\right)-I\;E\left[\Gamma_{i}\left(W\right)\right]\right).$$

Next, consider the class of functions

$$G_{W,l}=\left\{\left(z,v\right)\mapsto \left(1_{v\leq \alpha (t_{k}|U)}-1_{v\leq \delta_{j}+\alpha \left(t_{k}|U\right)}\right)W\;\left(\gamma^{-1}d\left(U,z\right)\right):\;d_{n,l-1}\leq \gamma \leq d_{n,l}\right\}.$$

Consequently, we obtain

$$\begin{array}{c} I\;P\;\left\{\underset{A_{n}\leq d_{n}\leq B_{0}}{\sup}\underset{|\delta |\leq M}{\sup}\underset{t\in [0,1]}{\sup}\left(\sqrt{\frac{nP_{U}\left(d_{n}\right)}{\log n}}\left|V_{n}\left(t_{k},\delta_{j},d_{n}\right)-I\;E\left[V_{n}\left(t_{k},\delta_{j},d_{n}\right)\right]\right|\right)\geq \eta_{0}\right\}\\ \;\leq \sum_{k=1}^{l_{n}}\sum_{j=1}^{l_{n}}\sum_{l=1}^{L(n)}I\;P\;\left\{\frac{1}{\sqrt{nP_{U}\left(d_{n,l}/2\right)\log n}}{∥\sqrt{n}\vartheta_{n}\left(W\right)∥}_{G_{W,l}}\geq \eta_{0}\right\}\\ \;\leq l_{n}^{2}\;L\left(n\right)\;\max_{1\leq l\leq L(n)}I\;P\;\left\{\max_{1\leq k\leq n}{∥\sqrt{k}\vartheta_{k}\left(W\right)∥}_{G_{W,l}}\geq \eta_{0}\sqrt{nP_{U}\left(d_{n,l}/2\right)\log n}\right\}. \end{array}$$

So all it remains to evaluate the term

$$I\;P\left\{\max_{1\leq k\leq n}{∥\sqrt{k}\vartheta_{k}\left(W\right)∥}_{G_{W,l}}\geq \eta_{0}\sqrt{nP_{U}\left(d_{n,l}/2\right)\log n}\right\}.$$

To this end, we apply Bernstein’s inequality for empirical processes, which necessitates the study of the asymptotic behavior of the following quantities.

$$\beta_{n}=I\;E\left[∥\sqrt{n}\vartheta_{n}{\left(W\right)∥}_{G_{W,l}}\right]\;\mathrm{and}\;\sigma^{2}=I\;E\left[G_{W,l}^{2}\left(U\right)\right],$$

where $G_{W,l}$ denotes the envelope function of the class $G_{W,l}$. Firstly, observe that, from condition (8), the envelope function $G_{W,l}$ satisfies

$$G_{W,l}\left(z\right)\leq C\;1_{B(U,d_{n,l}/2)}\left(z\right).$$

Consequently, for every p≥1,

(A1) $$I\;E\;\left[G_{W,l}^{p}\left(U\right)\right]\leq C^{p}\;I\;E\;\left[1_{B(U,d_{n,l}/2)}\right]\leq C^{p}P_{U}\left(d_{n,l}/2\right),$$

and in particular for p=2 we obtain

$$\sigma^{2}=I\;E\left[G_{W,l}^{2}\left(U\right)\right]=O\left(P_{U}\left(d_{n,l}/2\right)\right).$$

We now turn to the term $\beta_{n}$. By using the entropy assumption (7) we obtain

$$I\;E\left[∥\vartheta_{n}{\left(W\right)∥}_{G_{W,l}}\right]\leq CJ\left(1,W\right){∥F∥}_{2},$$

where *F* denotes the envelope function of the class W. Hence,

$$I\;E∥\vartheta_{n}\left(W\;1_{B(U,d_{n,l}/2)}\right){∥}_{G_{W,l}}\leq 2CJ\left(1,W\right){∥F\;1_{B(U,d_{n,l}/2)}∥}_{2}.$$

As a consequence, we deduce that

$$I\;E\;\left[∥\sqrt{n}\vartheta_{n}{\left(W\right)∥}_{G_{W,l}}\right]\leq C\sqrt{nP_{U}\left(d_{n,l}/2\right)}.$$

We are now in a position to apply Bernstein’s inequality (see Lemma A1) with

$$\beta_{n}=O\;\left(\sqrt{nP_{U}\left(d_{n,l}/2\right)}\right),\;\sigma^{2}=O\;\left(P_{U}\left(d_{n,l}/2\right)\right),\;H=C,$$

and

$$t=\frac{\eta_{0}}{2}\sqrt{nP_{U}\left(d_{n,l}/2\right)\log n}.$$

This yields

$$\begin{array}{c} I\;P\left\{\max_{1\leq k\leq n}{∥\sqrt{k}\vartheta_{k}\left(W\right)∥}_{G_{W,l}}>\eta_{0}\sqrt{nP_{U}\left(d_{n,l}/2\right)\log n}\right\}\\ \;\leq I\;P\left\{\max_{1\leq k\leq n}{∥\sqrt{k}\vartheta_{k}\left(W\right)∥}_{G_{W,l}}>\beta_{n}+t\right\}\\ \;\leq \exp\;\left\{-\eta_{0}^{2}\frac{\log n}{8+C\sqrt{\frac{\log n}{nP_{U}\left(d_{n,l}/2\right)}}}\right\}\\ \;\leq n^{-C^{\prime }\eta_{0}^{2}}. \end{array}$$

Moreover, since L(n)≤2logn, we obtain

$$nL\left(n\right)\max_{1\leq j\leq L(n)}I\;P\left\{\max_{1\leq k\leq n}{∥\sqrt{k}\vartheta_{k}\left(W\right)∥}_{G_{W,l}}>\eta_{0}\sqrt{nP_{U}\left(d_{n,l}/2\right)\log n}\right\}\leq C\left(\log n\right)n^{1--C^{\prime }\eta_{0}^{2}}.$$

Choosing $\eta_{0}$ such that $C^{\prime }\eta_{0}^{2}>2$ yields

$$\underset{A_{n}\leq d_{n}\leq B_{n}}{\sup}\left|V_{n}\left(t_{k},\delta_{j},d_{n}\right)-I\;E\left[V_{n}\left(t_{k},\delta_{j},d_{n}\right)\right]\right|=O_{a.co.}\;\left(\sqrt{\frac{\log n}{nP_{U}\left(A_{n}\right)}}\right).$$

We now consider the first term. Observe that

$$\underset{|\delta |\leq M}{\sup}\underset{t\in [0,1]}{\sup}\left|V_{n}\left(t,\delta ,d_{n}\right)-V_{n}\left(t,\delta_{j(\delta )},d_{n}\right)\right|\leq \frac{1}{nP_{U}\left(d_{n}\right)}\sum_{i=1}^{n}Z_{i}^{0},$$

where

$$Z_{i}^{0}=\psi \left(V_{i}\right)W_{i},\;\psi \left(V_{i}\right)=\underset{|\delta |\leq M}{\sup}\underset{t\in [0,1]}{\sup}1_{\left\{|V_{i}-\delta_{j(\delta )}-\alpha \left(t|U\right)|\leq Cl_{n}\right\}}.$$

Since

$$I\;E\left[Z_{i}^{0}\right]=O\left(l_{n}\right)=o\;\left(\sqrt{\frac{\log n}{nP_{U}\left(A_{n}\right)}}\right),$$

the analysis of the term $\sup_{|\delta |\leq M}\sup_{t\in [0,1]}\left|V_{n}\left(t,\delta ,d_{n}\right)-V_{n}\left(t,\delta_{j(\delta )},d_{n}\right)\right|$ reduces to proving the intermediate result

$$\underset{A_{n}\leq d_{n}\leq B_{n}}{\sup}\left(\frac{1}{nP_{U}\left(d_{n}\right)}\left|\sum_{i=1}^{n}\left(Z_{i}^{0}-I\;E\left[Z_{i}^{0}\right]\right)\right|\right)=O_{a.co.}\;\left(\sqrt{\frac{\log n}{nP_{U}\left(A_{n}\right)}}\right).$$

Once again this last is obtained by employing the Bernstein’s inequality for empirical processes. To do that, we define

$$T_{n}\left(W\right)=\frac{1}{\sqrt{n}}\sum_{i=1}^{n}\left(Z_{i}^{0}-I\;E\left[Z_{i}^{0}\right]\right).$$

We the following class of functions

$$G_{W,l}^{\prime }=\left\{\left(z,v\right)\mapsto \psi \left(v\right)\;W\;\left(\gamma^{-1}d\left(U,z\right)\right)\;:\;d_{n,l-1}\leq \gamma \leq d_{n,l}\right\}.$$

It is straightforward to see that this class admits an envelope function $F_{W,l}\left(\cdot ,\cdot \right)$ satisfying

$$F_{W,l}\left(z,v\right)\leq C\;1_{B(U,d_{n,l}/2)}\left(z\right).$$

Using assumptions (8) and (9), we obtain

(A2) $$I\;E\left[F_{W,l}^{p}\left(U,V\right)\right]\leq C\;P_{U}\left(d_{n,l}/2\right),\;I\;E\left[F_{W,l}^{2}\left(U,V\right)\right]=O\;\left(P_{U}\left(d_{n,l}/2\right)\right).$$

Next, for the term $\beta_{n}^{\prime }=E\left[∥T_{n}{\left(W\right)∥}_{G_{W,l}^{\prime }}\right]$, we use entropy assumption (7) to obtain

$$I\;E\left[∥T_{n}{\left(W\right)∥}_{G_{W,l}^{\prime }}\right]\leq CJ\left(1,W\right){∥F∥}_{2},$$

it follows that

$$I\;E∥T_{n}\left(W\;1_{B(U,d_{n,l}/2)}\right){∥}_{G_{W,l}^{\prime }}\leq 2CJ\left(1,W\right){∥F\;1_{B(U,d_{n,l}/2)}∥}_{2}.$$

Thus

$$\beta_{n}^{\prime }=I\;E\;\left[∥\sqrt{n}\alpha_{n}{\left(W\right)∥}_{G_{W,l}^{\prime }}\right]=O\;\left(\sqrt{nP_{U}\left(d_{n,l}/2\right)}\right).$$

Applying Bernstein’s inequality to the empirical process $T_{n}\left(W\right)$ yields

$$\underset{|\delta |\leq M}{\sup}\underset{t\in [0,1]}{\sup}\underset{A_{n}\leq d_{n}\leq B_{0}}{\sup}\left|V_{n}\left(t,\delta ,d_{n}\right)-V_{n}\left(t,\delta_{j(\delta )},d_{n}\right)\right|=O_{a.co.}\;\left(\sqrt{\frac{\log n}{nP_{U}\left(A_{n}\right)}}\right).$$

Concerning the second term, it is also based on the application of Bernstein’s inequality for empirical processes, allowing us to obtain

$$\underset{A_{n}\leq d_{n}\leq B_{0}}{\sup}\underset{|\delta |\leq M}{\sup}\underset{t\in [0,1]}{\sup}\left|V_{n}\left(t,\delta_{j(\delta )},d_{n}\right)-V_{n}\left(t_{k(t)},\delta_{j(\delta )},d_{n}\right)\right|=O_{a.co.}\;\left(\sqrt{\frac{\log n}{nP_{U}\left(A_{n}\right)}}\right).$$

Finally, the remaining terms are non-stochastic and follow from the stationarity assumption together with the fact that

$$I\;E\left[Z_{i}^{0}\right]=O\left(l_{n}\right).$$

Therefore

$$\underset{A_{n}\leq d_{n}\leq B_{0}}{\sup}\underset{|\delta |\leq M}{\sup}\underset{t\in [0,1]}{\sup}|I\;E\left[V_{n}\left(t_{k(t)},\delta_{j(\delta )},d_{n}\right)\right]-I\;E\left[V_{n}\left(t_{k(t)},\delta ,d_{n}\right)\right]|=O\left(l_{n}\right)=o\;\left(\sqrt{\frac{\log n}{nP_{U}\left(A_{n}\right)}}\right),$$

and similarly

$$\underset{A_{n}\leq d_{n}\leq B_{0}}{\sup}\underset{|\delta |\leq M}{\sup}\underset{t\in [0,1]}{\sup}|I\;E\left[V_{n}\left(t_{k(t)},\delta ,d_{n}\right)\right]-I\;E\left[V_{n}\left(t,\delta ,d_{n}\right)\right]|=O\left(l_{n}\right)=o\;\left(\sqrt{\frac{\log n}{nP_{U}\left(A_{n}\right)}}\right).$$

We now turn to the bias term. For all t∈(0,1) and δ∈[−M,M], we write

$$\begin{array}{ccc} I\;E[V_{n}\left(t,\delta ,d_{n}\right)] & = & -\frac{1}{P_{U}\left(d_{n}\right)}I\;E\;\left[\left(1_{\left\{V_{1}\leq \delta +\alpha \left(t|U\right)\right\}}-1_{\left\{V_{1}\leq \alpha \left(t|U\right)\right\}}\right)W_{1}\right]\\ & = & -\frac{1}{P_{U}\left(d_{n}\right)}I\;E\;\left[\left(\mathrm{Cdf}(\delta +\alpha (t|U)|U)-\mathrm{Cdf}(\alpha (t|U)|U)\right)W_{1}\right]\\ & = & -\frac{1}{P_{U}\left(d_{n}\right)}I\;E\;\left[\left(\mathrm{Cdf}(\delta +\alpha (t|U)|U)-\mathrm{Cdf}(\alpha (t|U)|U)\right)W_{1}\right]+O\left(a_{n}^{b}\right)\\ & = & -\frac{\delta \;Pd(\alpha (t|U)|U)}{I\;E[W_{1}]}I\;E\left[W_{1}\right]+O\left(d_{n}^{b}\right)+o\left(\delta \right). \end{array}$$

□
